# Supplementary material for: “Anees Has Measles”: Storytelling and Singing to Enhance MMR Vaccination in Child Care Centers Amid Religious Hesitancy
Source: Vaccines (Basel). 2024 Jul 22;12(7):819. doi: 10.3390/vaccines12070819 (PMC11281558; doi:10.3390/vaccines12070819)
Supplement: Supplementary file 1 [file vaccines-12-00819-s001.zip › vaccines-3014519-supplementary.pdf]

อานีส
انيس
เป็นหัด
مغهيدڤي چمڤق

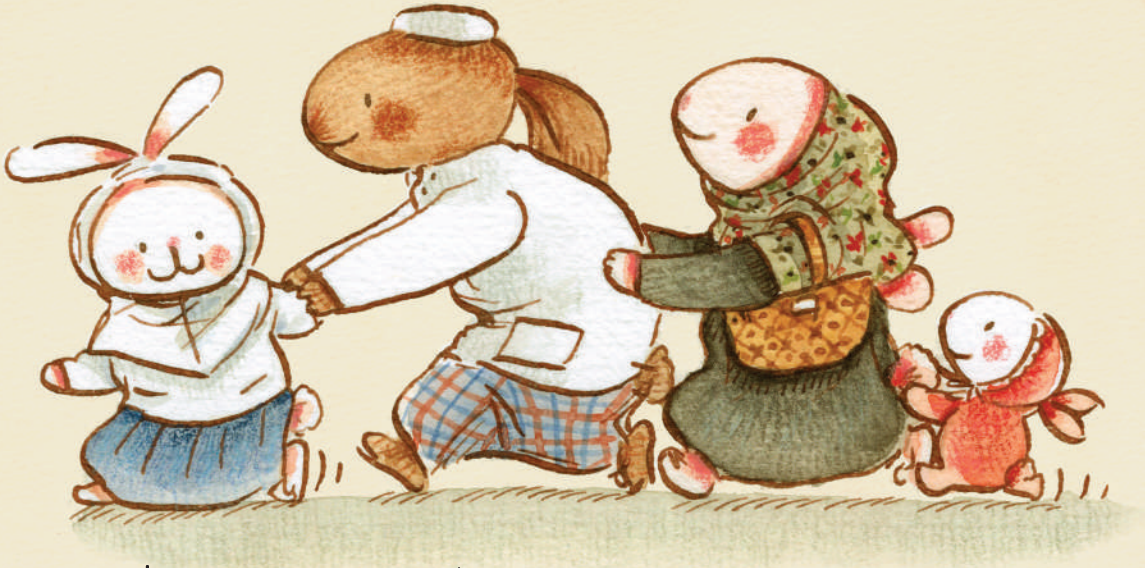

ฉันชื่ออานีส นี่คือป๊ะ มะ กับน้องนิน

نام ساي انيس. اين ڤاڤا، ماما دان اديق ساي.

อานีสตื่นเช้า   กินข้าวเร็วไว

انيس باڠون ڤاڬي، ماكن ناسي چڤت٢.

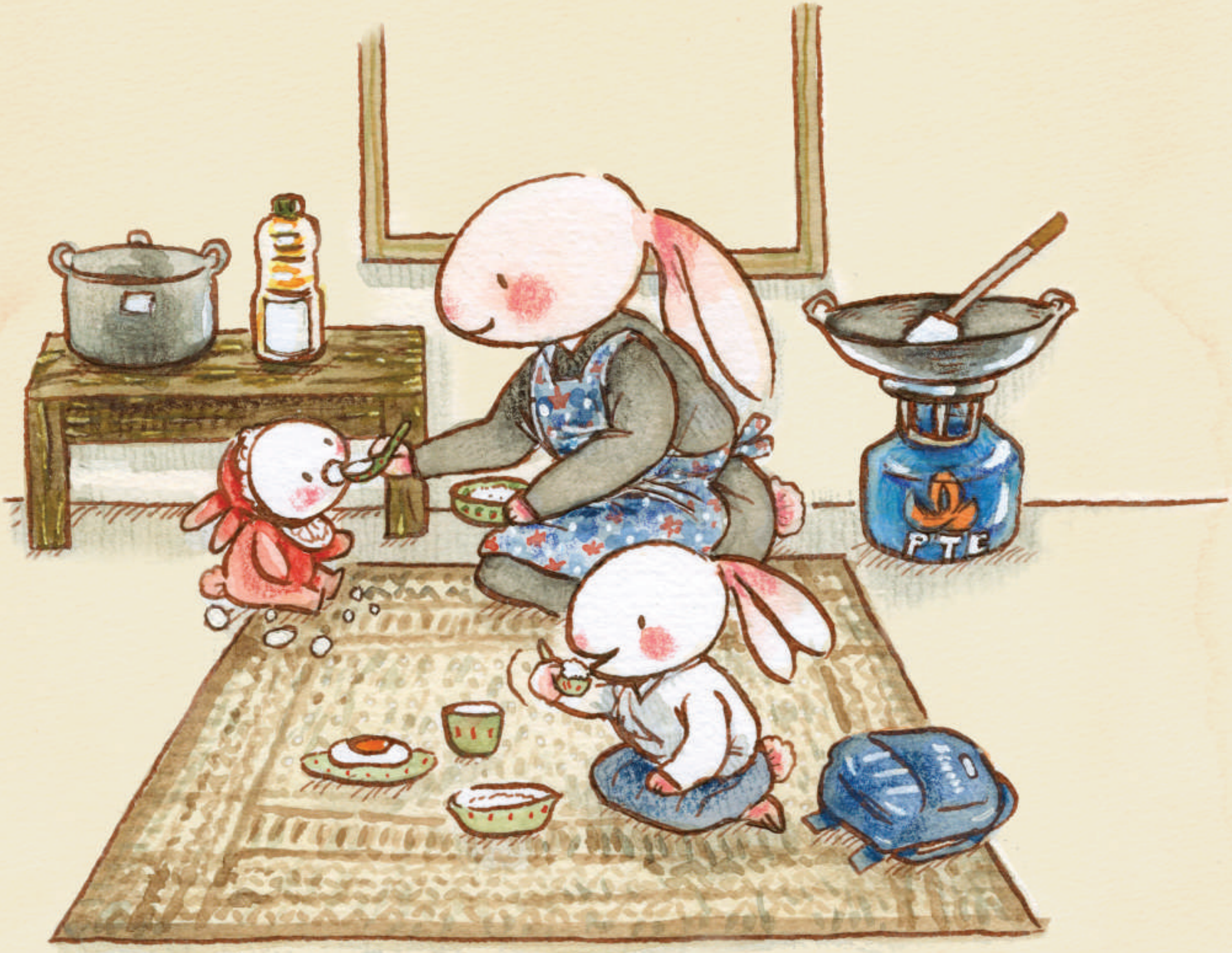

วันนี้แจ่มใส   ได้ไปโรงเรียน

هاري اين سڬرهاتي،   انيس داڤت ڤرڬي سکوله

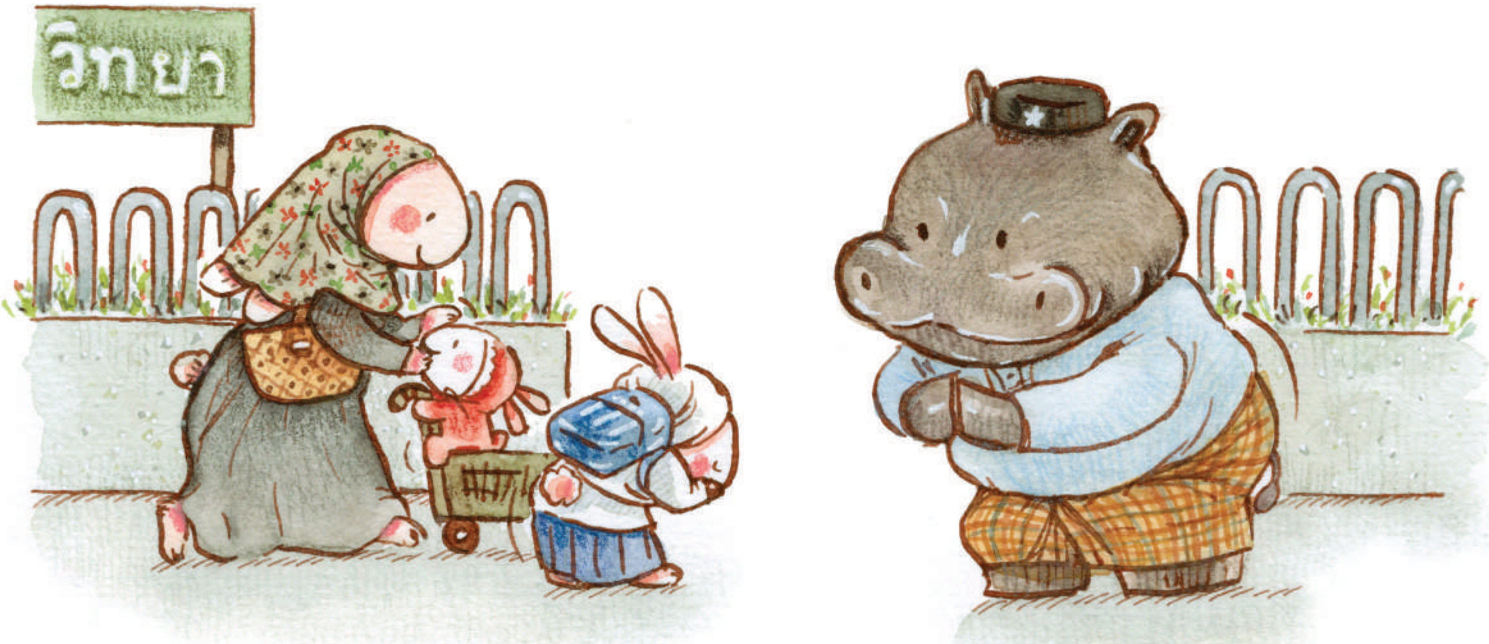

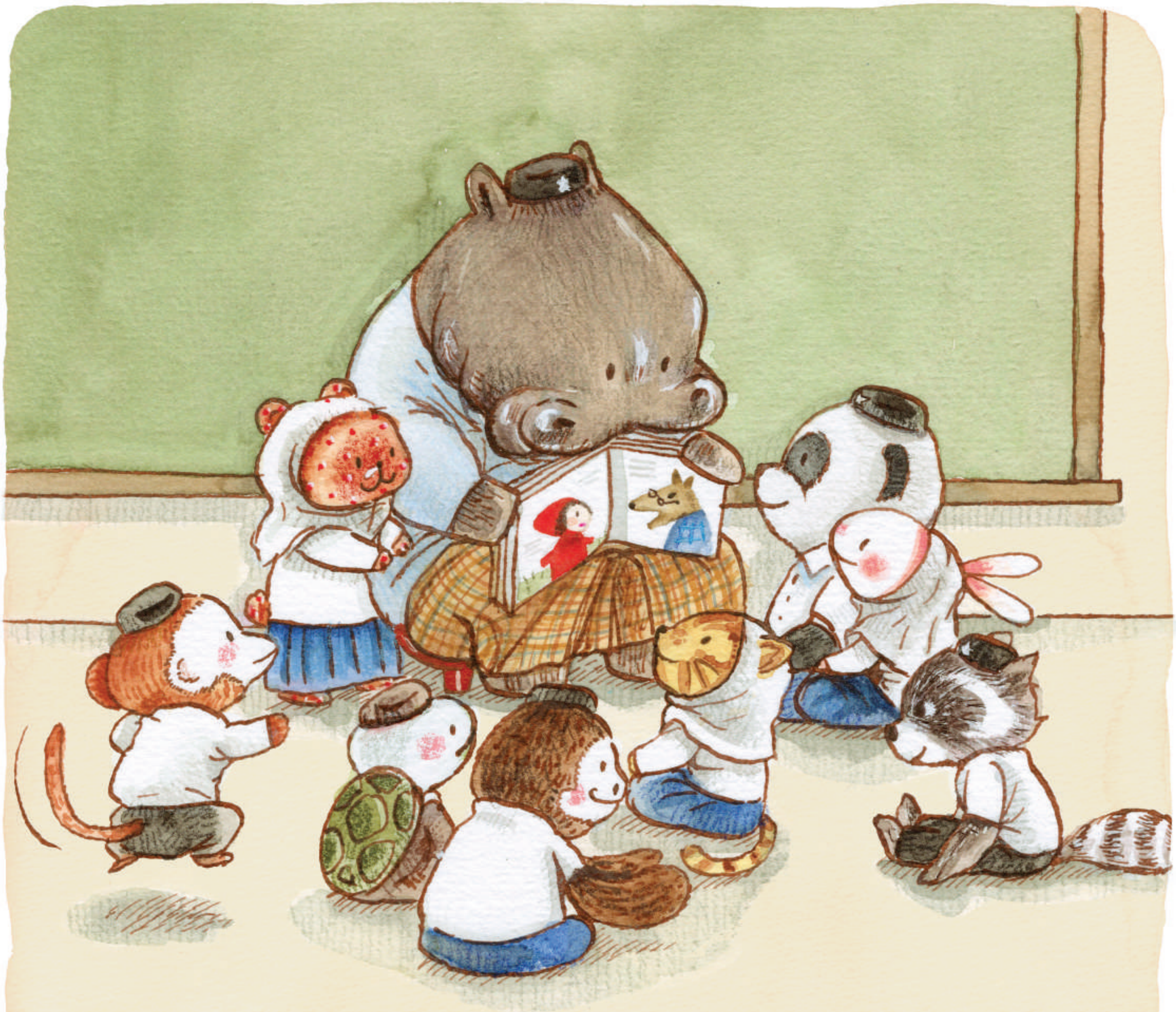

ครูอ่านนิทาน เบิกบานสุขใจ

چيقݢو ممباچ بوكو چريتا، انيس براس رياڠ ݢمبيرا .

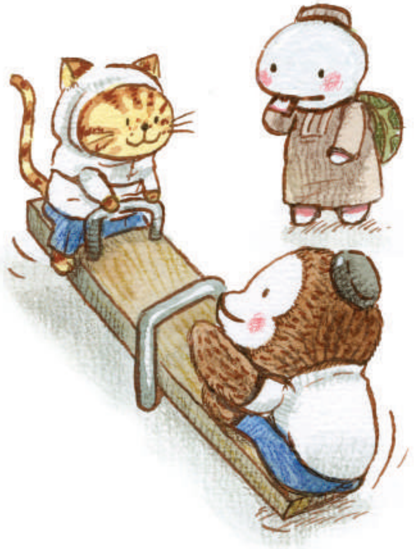

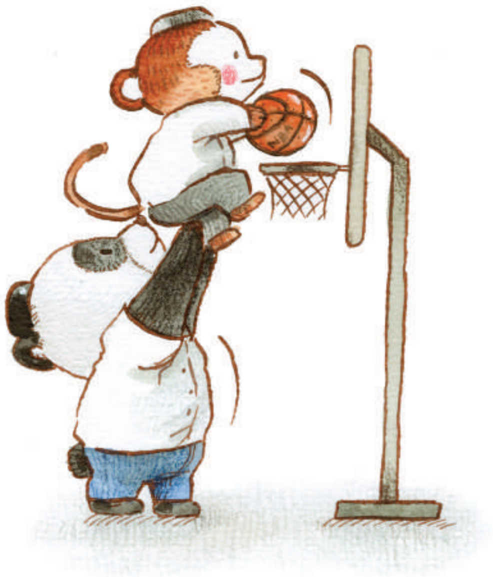

จบแล้วค่อยไป   เล่นในสนาม

سلڤس مندڠر چريتا، ڤرڬي مائين دڤادڠ ڤولا.

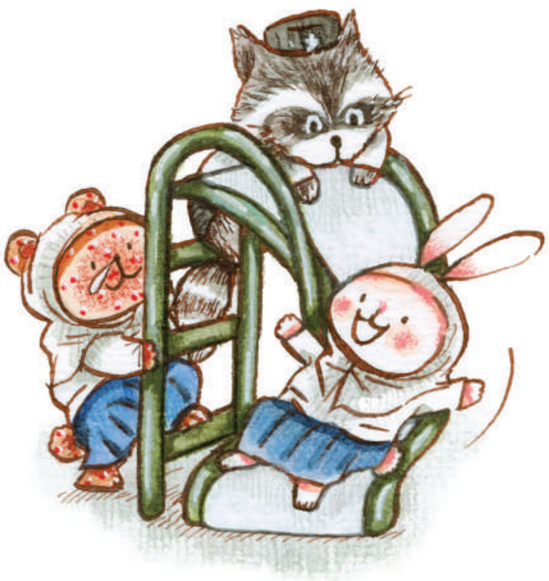

อานีสจับมือ
เขย่งเท้าขึ้นมา

กอล์ฟถือตุ๊กตา
นั่งชิงช้ากัน

انيس ڤڬڠ تاڠن،
جينجيڠ كاكي تيڠڬي٢،

قاف ڤڬڠ بونيكا.
دودوق اتس بواين.

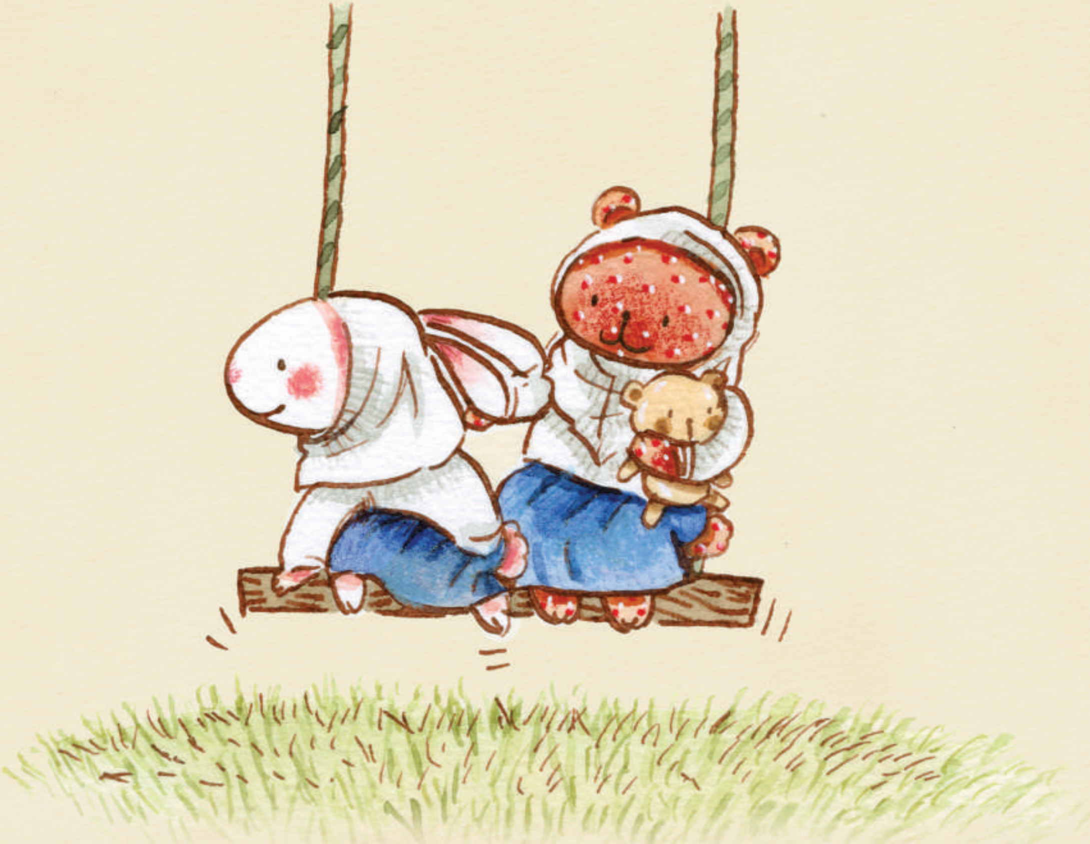

อานีสกอดคอ หัวเราะสนุก
กอล์ฟจามไม่หยุด ฮัดเช้ย ฮัดเช้ย

انيس ڤلوق ليهير، كتاوا ريا.
قاف اشيق برسين هتچو ...هتچو....هتچو ...

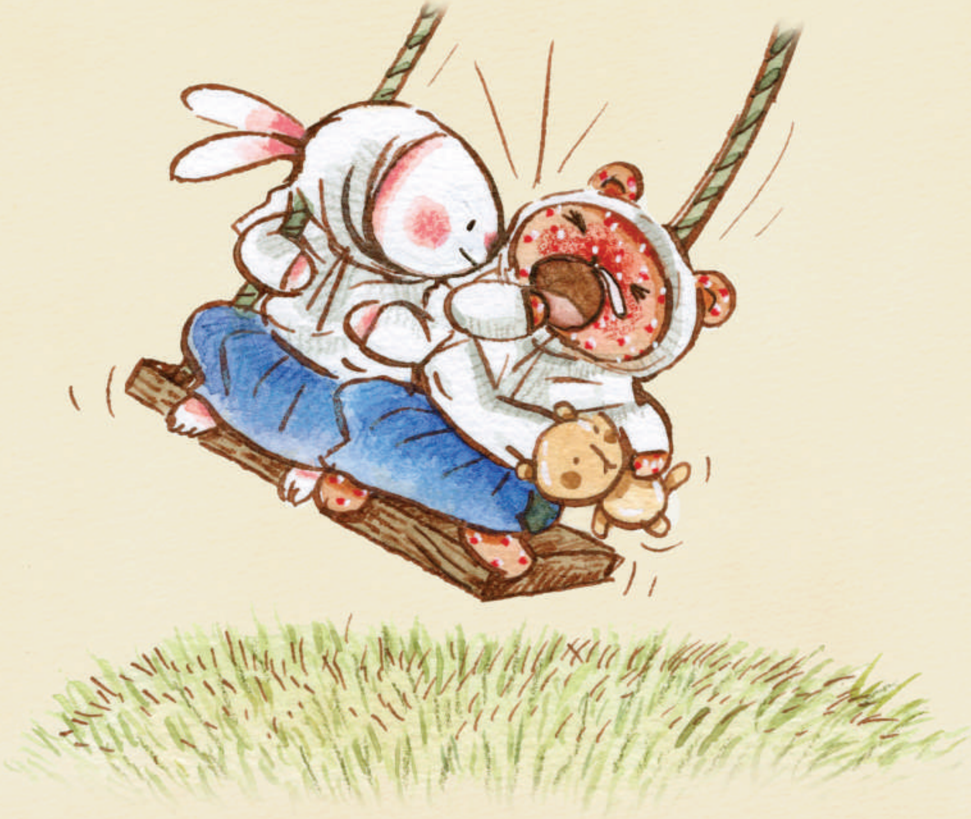

สิบวันผ่านไป อานีสมีไข้
ไปเรียนไม่ไหว ทั้งไอทั้งจาม

سڤولوه هاري کمودين انيس براس دمم.
تق داڤت ڤرݢي سکوله، باتوق، لاݢي برسين.

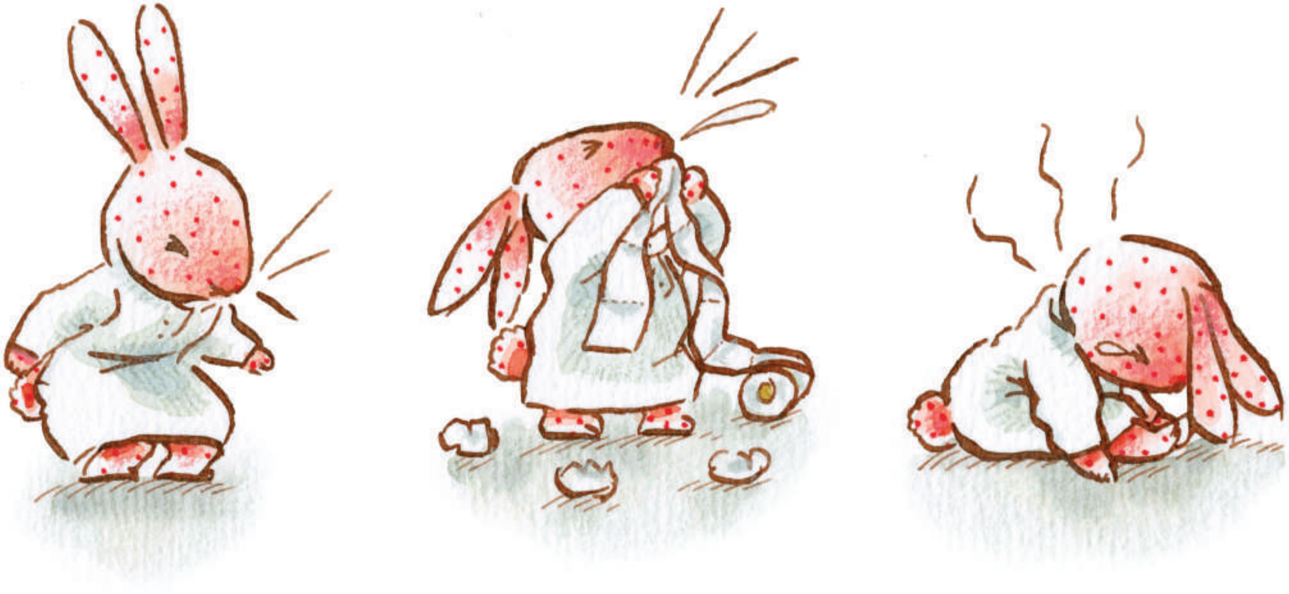

อานีสหมดแรง   หน้าแดงตัวรุม
ปากแห้งตาชุ่ม   มะอุ้มออกมา

انيس هيلڠ تناک،
مولوت کريڠ، مات براير.
موک ميره، ڤانس بادن،
ماما دوکوڠ انيس کلوار.

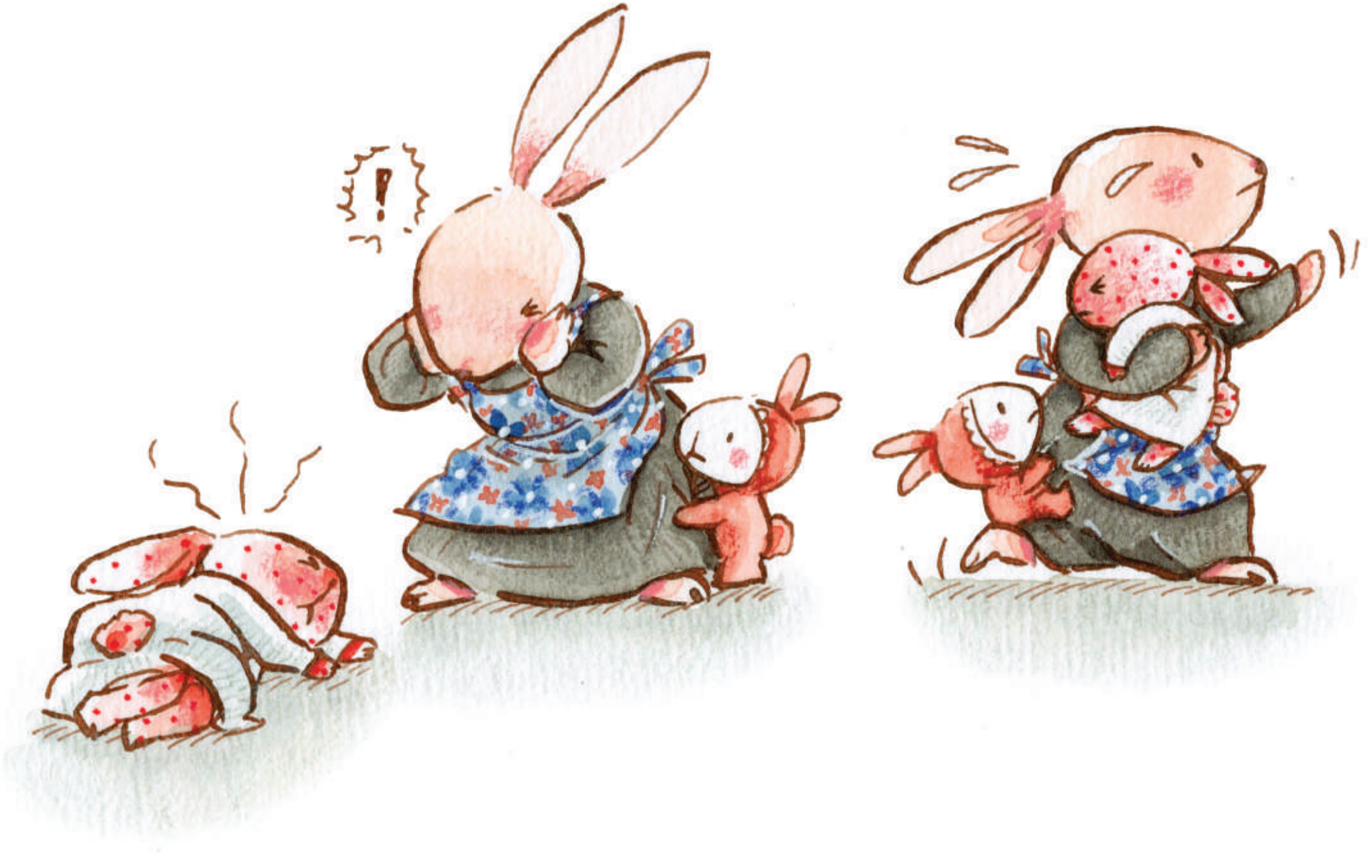

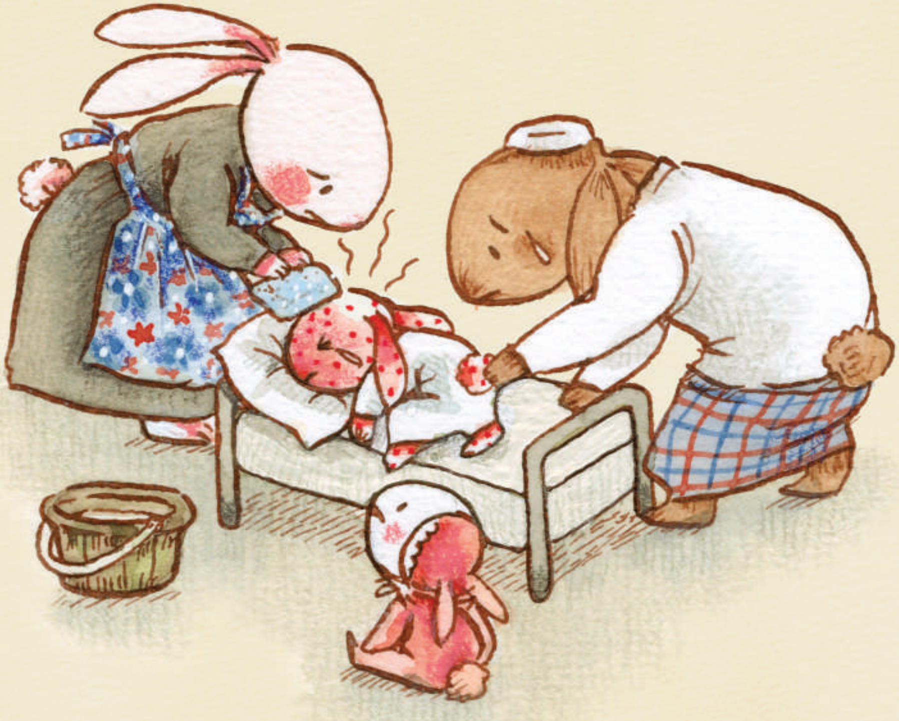

ป๊ะจ๋าดูหน่อย   ลูกน้อยเป็นไร

" ڤاڤا، ماريله تيڠوق،   انق جادي اڤ ني ؟"

มะรอไม่ไหว   ต้องไปหาหมอ

ماما تق صبر نق توغݢو،   باوا انيس جومڤا دوكتور.

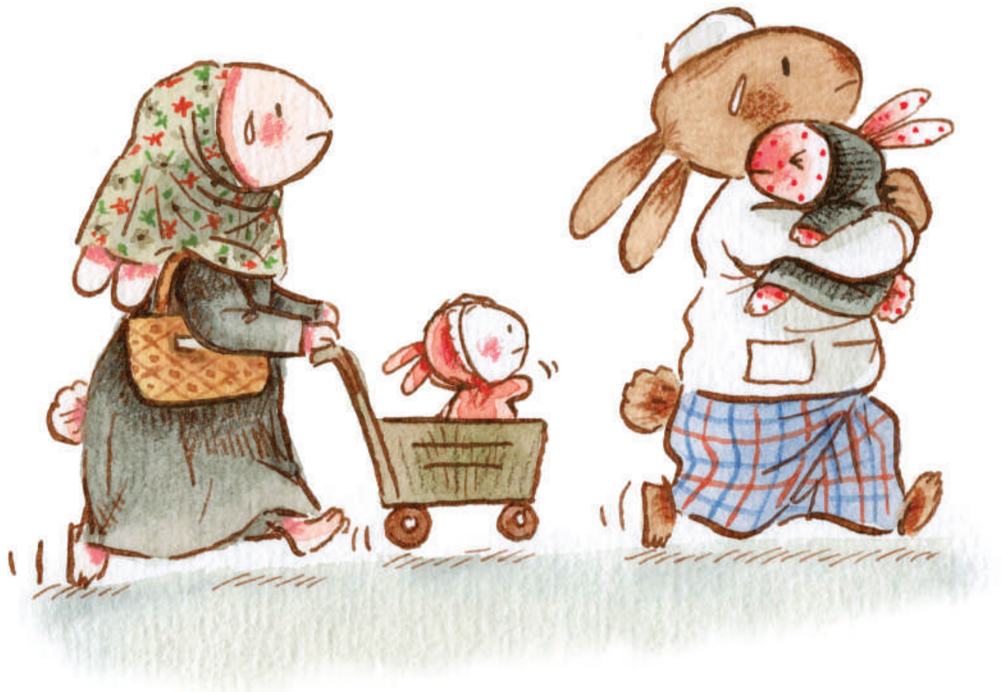

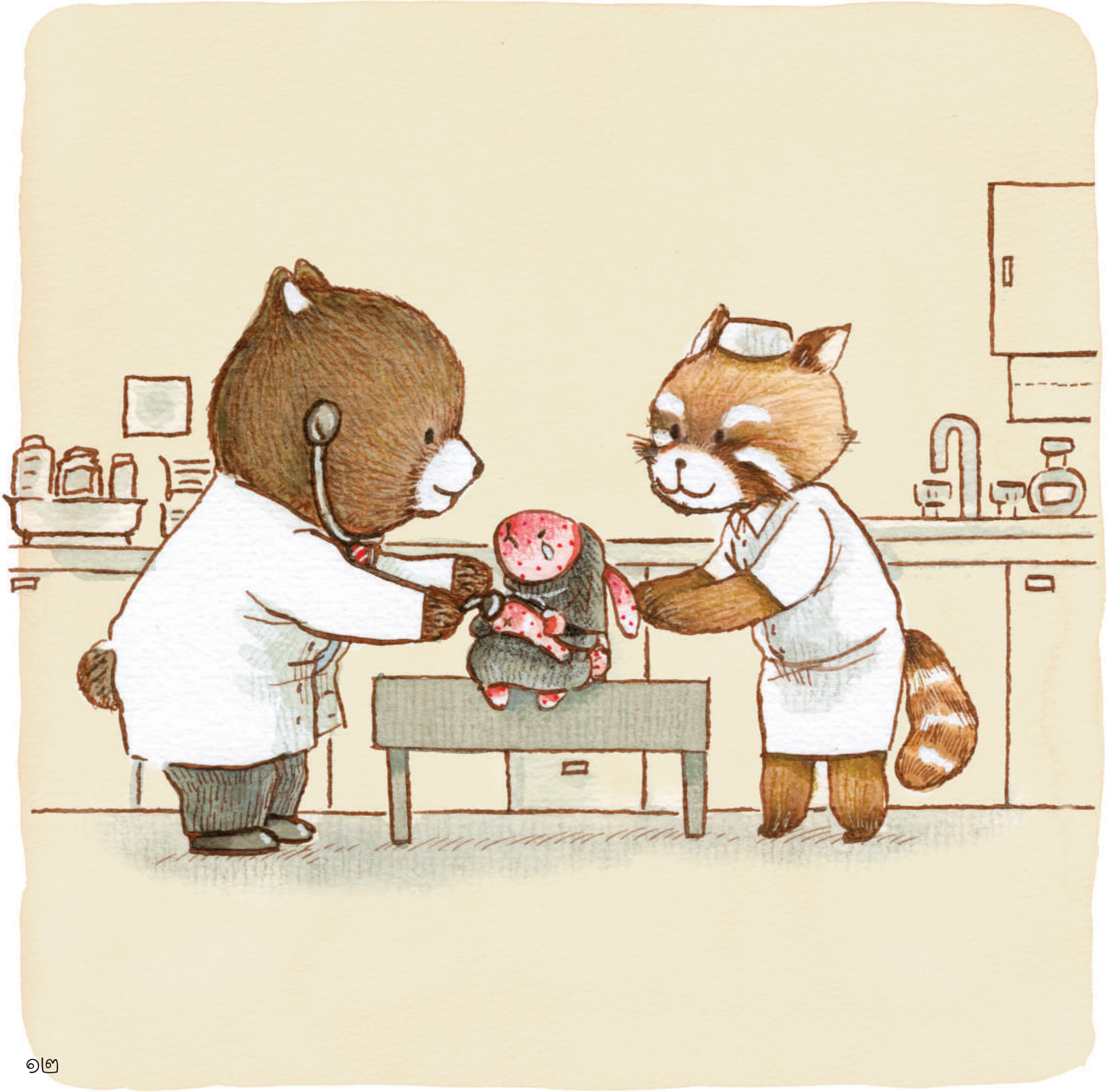

หมอตรวจอาการ
ดีที่รีบมา

بادن لالو بركات،
"انيس دمم چمڤق."

ป๊ะมะตกใจ
โรคหัดแบบนี้

"نق بواة ماچم مان ني ؟
اد اوبت تق؟"

ไม่นาน...บอกว่า
"อานีสเป็นหัด"

دوكتور ڤريقسا
موجور داتڠ چڤت،

ทำไงกันดี
หมอมียาไหม

ڤاڤا، ماما تركجوت،
ڤياكيت چمڤق ماچم اين

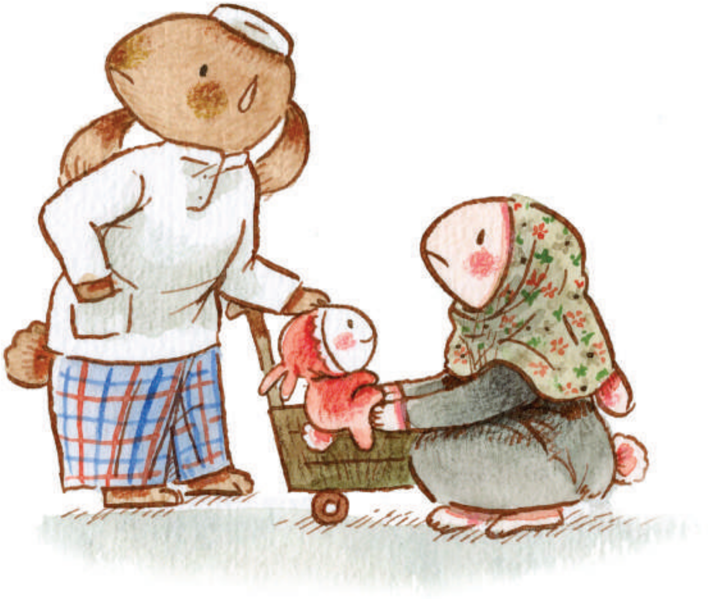

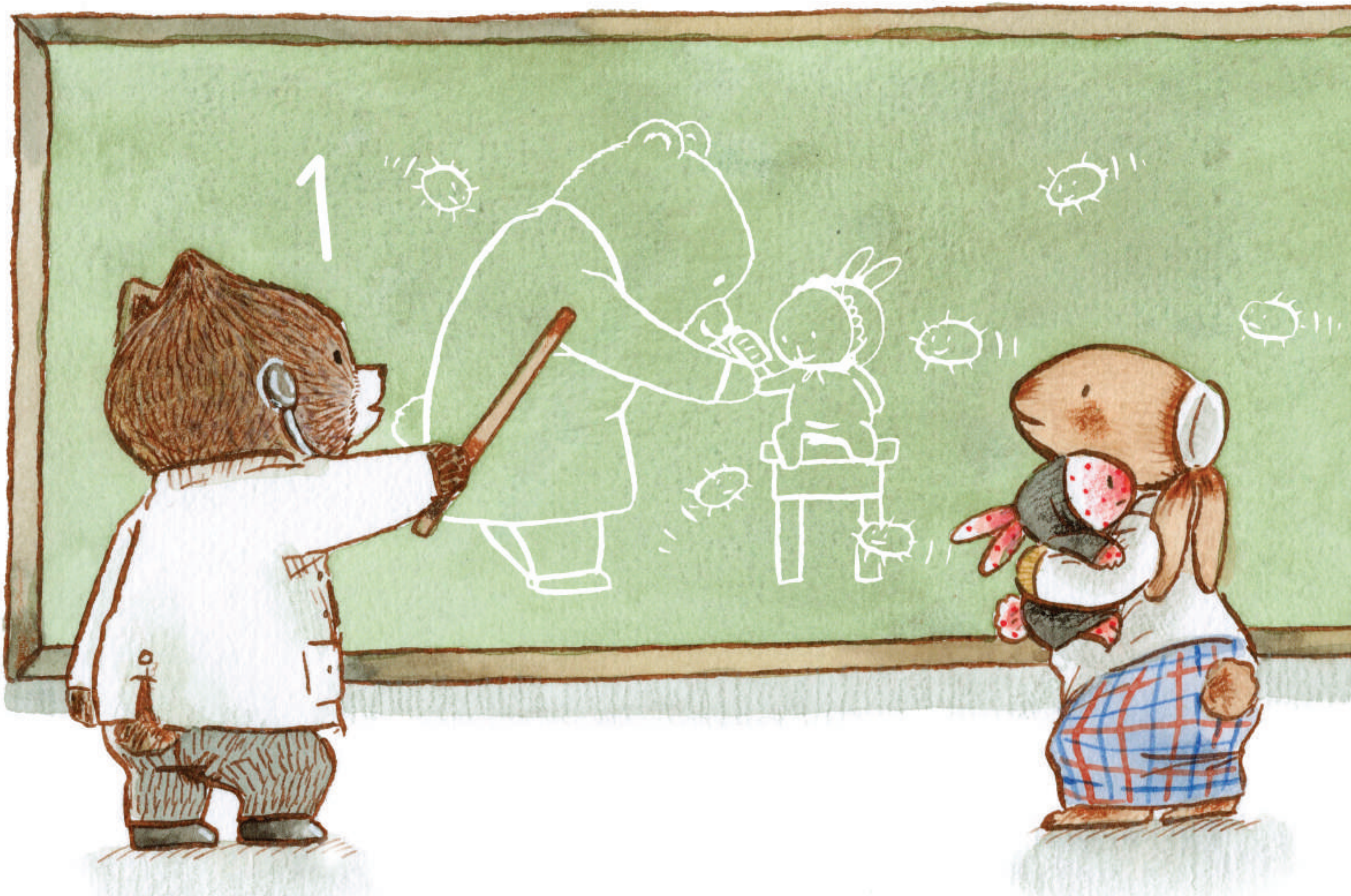

หมอต้องเฝ้าดู ให้รู้แน่ชัด
ไม่ใช่แค่ ‘หัด’ ไวรัสถึงปอด

دوكتور مستي توڬكو، بيار تاهو دڠن جلس.
بوكن سقدر چمڨق، ۏيروس بوليه سمڨاي كڤارو٢.

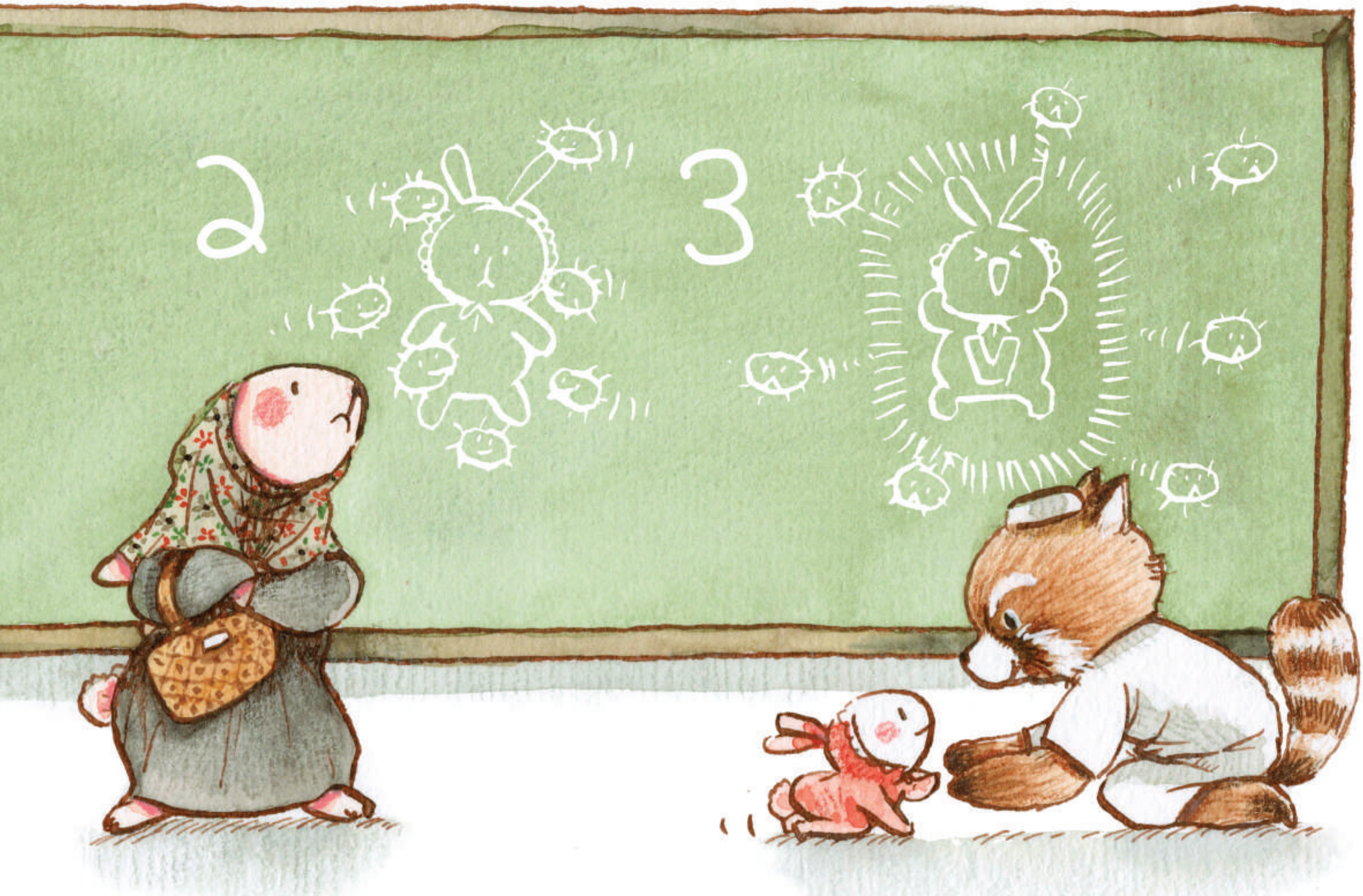

หัดแพร่ระบาด ประมาทไม่ได้
วัคซีนกันไว้ ฉีดให้น้องก่อน

چمڤق بوليه مريبق، جاڠنله چواي.
سونتيقكن ۏكسين، جاڬ اديق دولو.

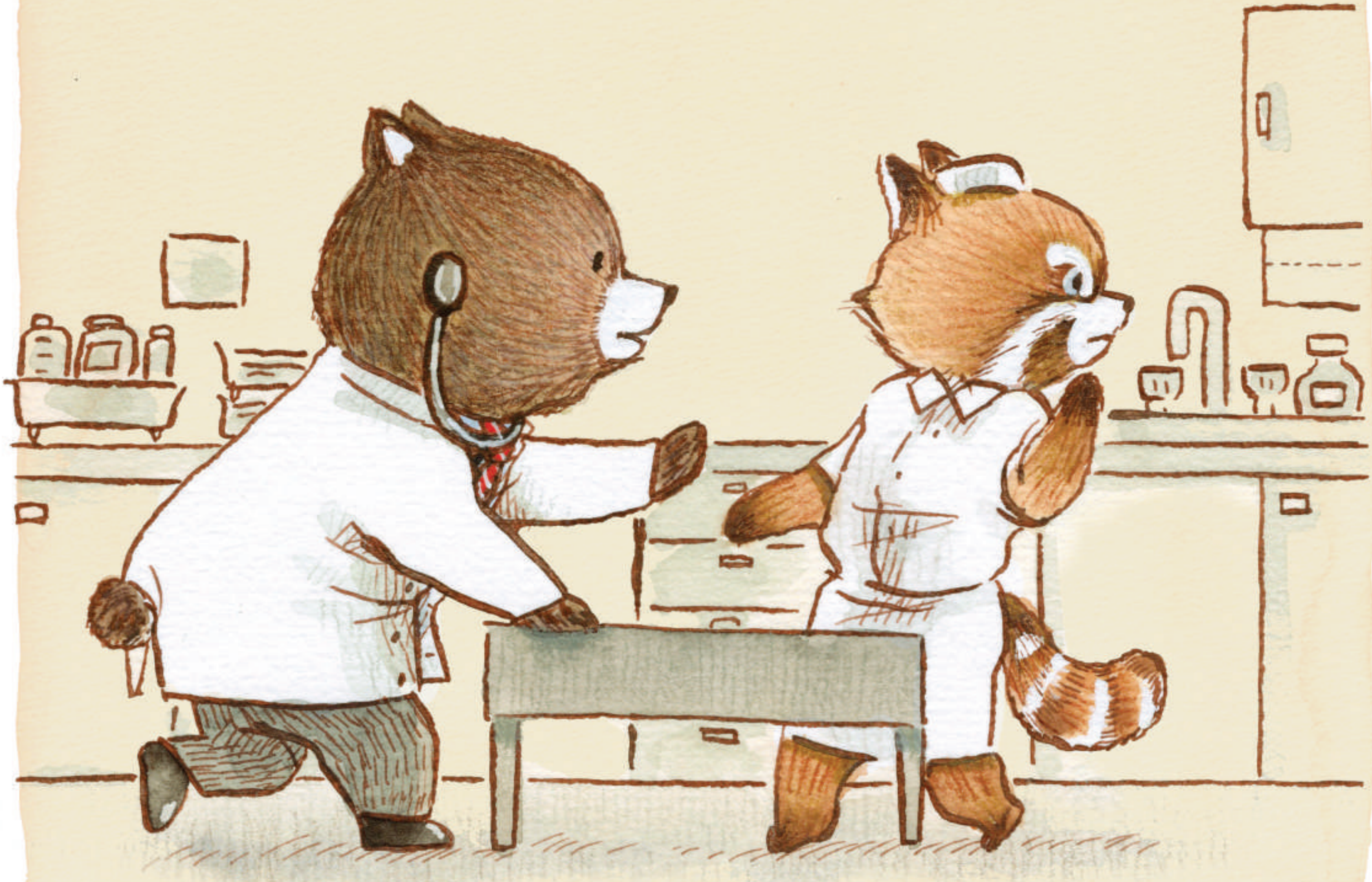

ออกหัดอันตราย หมอให้ยากิน
แนะนำน้องนิน ฉีดวัคซีนเลย

چمڤق بربهاي، دوكتور باݢي ماكن اوبت.
دوكتور منصيحتكن سوڤاي اديق نين دسونتيقكن ۏكسين.

ป๊ะมะตกใจ   ยาฉีดได้ไง
มันไม่ฮาลาล !!

ڤاڤا، ماما ترکجوت، "سونتيقن اوبت؟
ڤرکارا ايت تيدقله حلال."

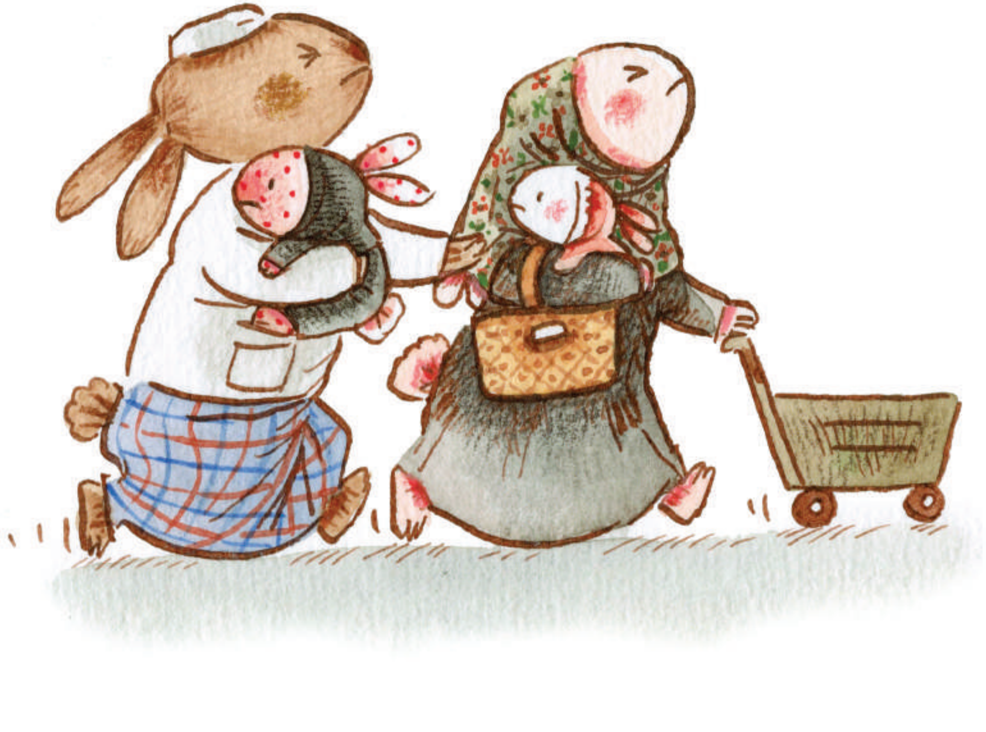

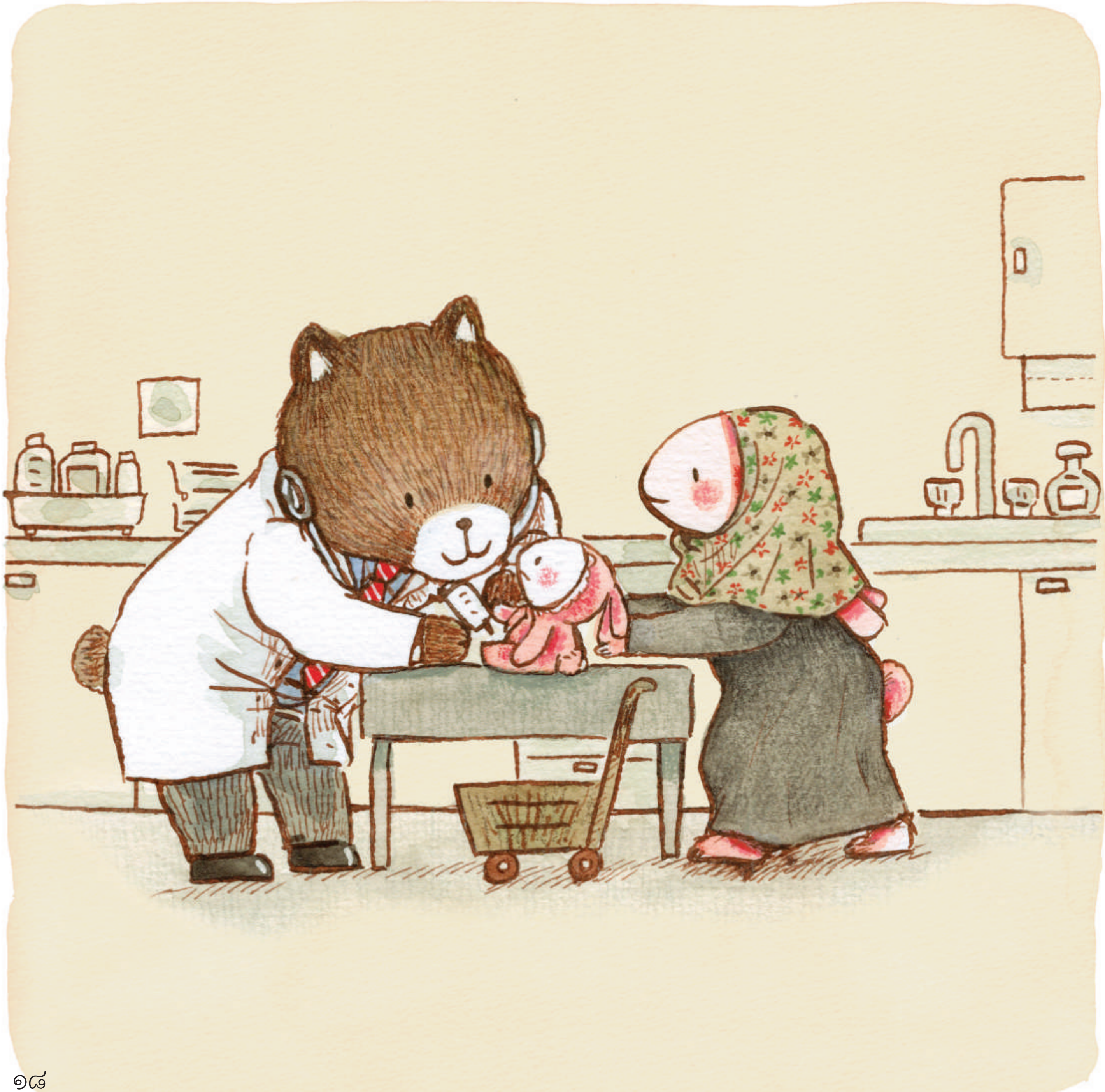

นบีกล่าวไว้ เมื่อใดคับขัน
ถือว่าสิ่งนั้น ฮาลาล ฮาลาล

نبي برسبدا اڤابيلا ضرورة،
ايت دأڠڬڤ حلال حلال.

ป๊ะบอกเต็มใจ ยอมให้นินมา
มะรีบพยักหน้า ฉีดยาวัคซีน

ڤاڤا ريلا، بيار نين داتڠ.
ماما مڠڠڬوق، بيار نين دسونتيقكن ۏكسين.

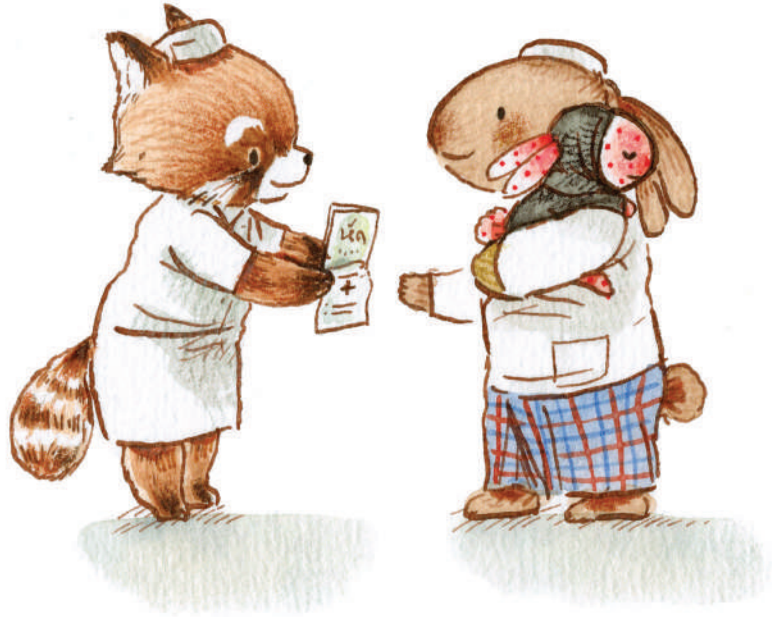

อานีสหายป่วย คุณหมอช่วยได้
และต้องจำไว้ ‘หัด’ ร้ายแรงมาก

انيس سوده سمبوه، دوكتور داڤت تولوڠ.
كيت ڤرلو ايڠت ”چمڤق“ ساڠت بربهاي.

ป๊ะบอกเพื่อนบ้าน ลูกหลานปลอดภัย
วัคซีนช่วยให้ ไม่ป่วยเป็นหัด

ڤاڤا ممبريتاهو جيرن، ” انق-چوچو اكن سلامت،
ۏكسين داڤت بنتو مڠيلقكن چمڤق.“

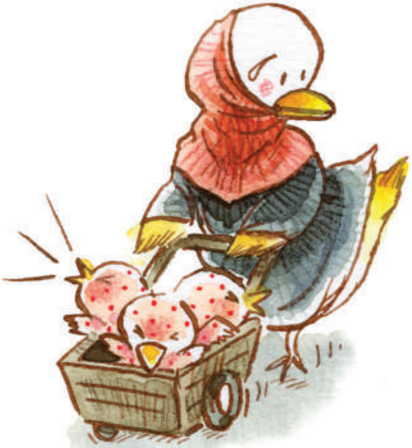

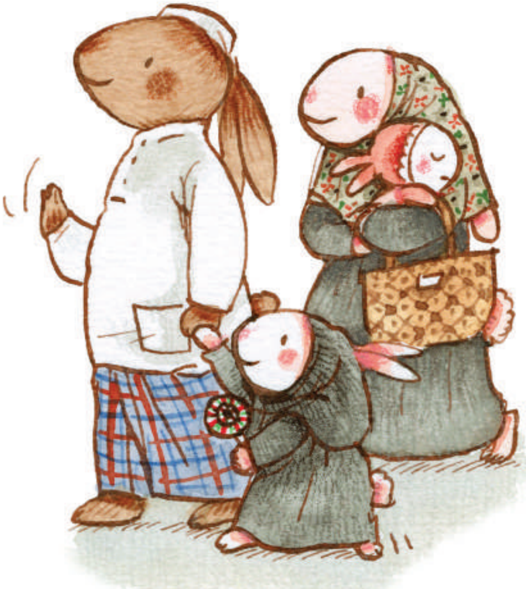

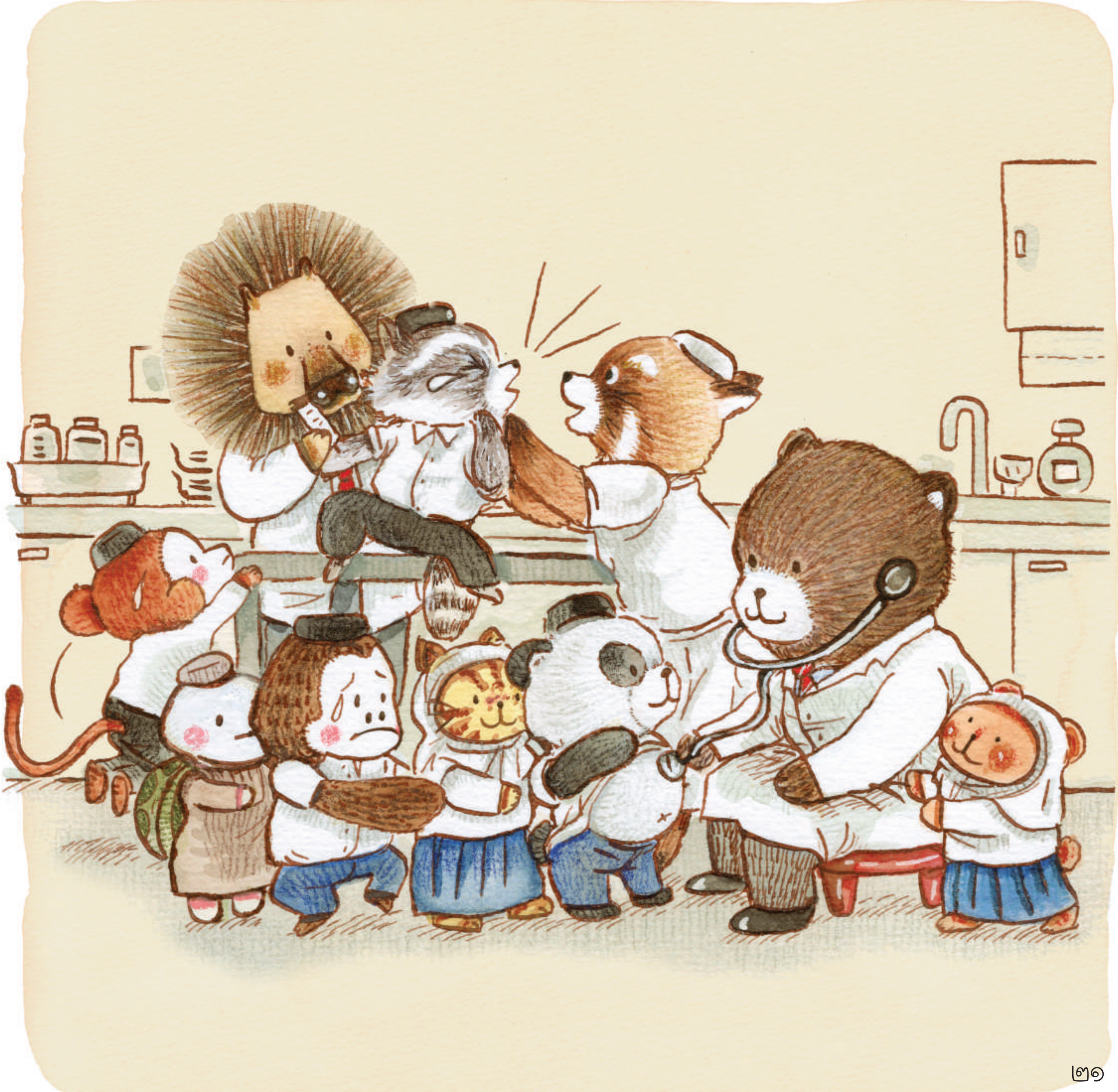

คุย สร้าง สุข เพื่อการอ่านสร้างสุข

โรคหัดเกิดจากเชื้อไวรัส แพร่ระบาดได้ง่ายผ่านการไอ จาม หรือพูดคุยกันในระยะใกล้ชิด มักพบการระบาดในที่รวมคนหมู่มาก เช่น ในศูนย์พัฒนาเด็กเล็ก โรงเรียน ชุมชนที่อยู่กันหนาแน่น ในทุกภูมิภาคของโลก เช่น ประเทศอินเดีย ยูเครน ฟิลิปปินส์ บราซิล พบผู้ป่วยจำนวนสูงสุด

ผู้รับเชื้อมักจะมีอาการเริ่มต้นคล้ายไข้หวัด มีไข้ มีน้ำมูก ไอแห้ง ๆ และเริ่มมีผื่นนูนแดง

ในช่วง ๒ ปีที่ผ่านมา สำนักระบาดวิทยาพบสถานการณ์ระบาดในพื้นที่ภาคใต้ตอนล่างของประเทศไทย ผู้ป่วยส่วนใหญ่เป็นเด็กก่อนวัยเรียนและเด็กนักเรียนที่ไม่ได้ฉีดวัคซีนป้องกันโรคหัด ประกอบกับภาวะทุพโภชนาการ (ภาวะจากการได้รับสารอาหารไม่เพียงพอ เกิน หรือผิดสัดส่วน) ทำให้เด็กมีอัตราการเสียชีวิตสูง เนื่องจากภาวะแทรกซ้อน

การป้องกันโรคหัดที่ได้ผล คือ นำเด็กไปรับวัคซีน ๒ ครั้ง เมื่ออายุ ๙ เดือน และ ๒ ปีครึ่ง ตามสถานบริการสาธารณสุขของรัฐทุกแห่งทั่วประเทศ

อานิสเป็นหัด เล่มนี้ เกิดขึ้นจากความห่วงใยถึงปัญหาที่เกิดขึ้น

ขอบคุณทีมนักศึกษาชั้นปีที่ ๔ (พ.ศ. ๒๕๖๒) คณะแพทยศาสตร์ มหาวิทยาลัยสงขลานครินทร์ ที่ได้ออกแบบสร้างสรรค์หนังสือจากรายวิชาสร้างเสริมสุขภาพ และได้นำไปทดลองใช้ที่โรงเรียนรัศมีสถาปนา อ.มายอ จ.ปัตตานี จนมีการเก็บข้อมูลเด็ก ๆ เพื่อร่วมป้องกันและแก้ไขปัญหาที่จะเกิดขึ้น

ขอบคุณ ผศ.นพ.เทอดพงศ์ ทองศรีราช ที่ชักชวนแผนงานสร้างเสริมวัฒนธรรมการอ่าน สสส. ให้นำต้นฉบับของนักศึกษามาพัฒนาต่อ รวมทั้งการระดมทุนจัดพิมพ์เพื่อขยายผลให้กว้างขวาง

และขอขอบคุณเป็นพิเศษต่อบรรณาธิการเล่ม คุณระพีพรรณ พัฒนาเวช กรรมการมูลนิธิสร้างเสริมวัฒนธรรมการอ่าน และคณะ ที่บรรจงสร้างสรรค์ พัฒนาเรื่องและภาพอย่างอ่อนโยน และเร่งให้ความสำคัญเรื่องเงื่อนเวลา เพื่อให้ทันต่อภาวการณ์ที่กำลังส่งผลกระทบในขณะนี้

สุดใจ พรหมเกิด

ผู้จัดการแผนงานสร้างเสริมวัฒนธรรมการอ่าน สสส.

بربوال ممبينا كبهاݢيائن اونتوق ڤمباچائن ممبينا كبهاݢيائن

ڤاكيت چمقق دسببكن اوليه كومن ۏيروس يڠ داڤت مريبق دڠن سڠ ملالوءي باتوق، برسين اتاو بربوال سچارا راڤت. ڤمريبقنڽ سريڠ برلاكو دكالڠن خلايق راماي سقرتي د ڤوست ڤمباڠونن كانق٢ كچيل دان سكوله. ترداڤت ڤاليڠ بايق جوملہ ڤساكيت د كومونيتي يڠ ڤست د ستياڤ رنتاو د دنيا سقرتي نݢارا ايندي، يوكرين، فيليڤينا دان برازيل.

اورڠ يڠمرنيما كومن ڤاكيت چمقق ممقوياءي ݢجالا اول سقرتي جوك دڠن دمم سلسما، يا ءيت دمم، برهيڠوس، باتوق كريڠ دان مولا بربدن ميره.

دالم تيمڤوه 2 تاهون يڠ لالو جابتن كاجي ڤمريبقن مناڤتي كمريبقن چمقق دكاوسن سلاتن بهاݢين باوه، نݢارا تايلاند. كباپقن ڤساكيت اداله كانق٢ يڠ بلوم برسكوله دان موريد٢ يڠ تيدق منريما سونتيقن ۏكسين اونتوق مڠيلقكن ڤاكيت چمقق، لاݢي ڤون ككورڠن ذات ماكنن يڠ ميببكن كانق٢ ممقوياءي قدر كماتين يڠ تيڠݢي عاقبة ڤاكيت سمقيڠن.

چارا مڠيلقكن ڤاكيت چمقق يڠ بركسن اياله ممباوا كانق٢ اونتوق منريما سونتيقن ۏكسين سباپق 2 كالي، سماس برعمور 9 بولن دان 2 تاهون ستڠه دستياڤ ڤجابت ڤرخدمتن كصيحتن كراجائن د سلوروه نݢارا.

بوكو انيس مڠهيدڤي چمقق اين دچيڤتا دري كبيمبڠن ترهادڤ مسئله يڠ تله ترجادي. تريما كاسيه كڤد ڤارا ڤلاجر تاهون 4 (2562 ب.) ، اونيۏرسيتي سوڠكلا ناكرين يڠ مريكابنتوق چيڤتائن بوكو دري مات ڤلاجرن ڤمبينائن كصيحتن دان بوكو اين تله دݢوناكن اونتوق ڤرچوبائن د سكوله راسمي ستاڤانا، دائيره مايور، ولايه ڤتتاني سهيڠڬ دأداكن ڤڠومڤولن داتا اونتوق مڠيلقكن دان ميلسايكن مسئله يڠ موڠكين برلاكو.

تريما كاسيه كڤد اسيستن ڤروفيسسور دوكتور تودڤوڠ طاڠسيرات يڠ مڠاجق اهلي رنچڠن ڤمبيناٴن بوديا مماچ اونتوق مماجوكن بوكو نسخه اصل ڤلاجرڽ، ترماسوق جوݢ ڤڠومڤولن درما اونتوق منچيڤتا دان مڽيباركن بوكو ايت.

تريما كاسيه سچارا خاص كڤد ڤونتيڠ بنتوق بوكو، انچيق راٴڤيڤن ڤتناويت، جاوتنكواس يايسن ڤمبيناٴن بوديا مماچ دان راكن - راكن يڠ منچيڤتا، مماجوكن چريتا دان ݢمبر دڠن بݢيتو سوڤن دان ممڤرچڤتكن كرجا سوڤاي داڤت دسلسايكن دالم جڠك ماس يڠ تڤت دڠن كأداٴن سماس.

سودچاي ڤرومكود
ڤڠاره رنچڠن ڤمبيناٴن بوديا مماچ

ชาวมุสลิมฉีดวัคซีนได้หรือไม่

"ความจำเป็น (ฎอรูเราะฮ์) มีผลทำให้สิ่งที่ฮารอมกลายเป็นสิ่งที่ฮาลาลได้"
ถอดความจากหลักการรุคเศาะฮฺ (การผ่อนปรน)
ในภาวะจำเป็นตามบทที่ ๒ อัลบะเกาะเราะฮฺ โองการที่ ๑๗๓

ปัจจุบันได้มีการยืนยันจากนักวิชาการมุสลิม ปราชญ์มุสลิม และท่านจุฬาราชมนตรี ซึ่งเป็นผู้นำของศาสนาอิสลามในประเทศไทยว่า ชาวมุสลิมสามารถฉีดวัคซีนป้องกันโรคได้ ทุกท่านให้ความเห็นตรงกันว่า การฉีดวัคซีน เป็นการดูแลร่างกายที่พระเจ้าประทานมาให้ตามคำสอนของพระเจ้า การไม่ฉีดวัคซีน นอกจากจะเป็นการทำร้ายตนเองแล้ว ยังเป็นการทำร้ายผู้อื่นอีกด้วย หากเราเป็นคนแพร่เชื้อถือเป็นการทำบาปอย่างหนึ่ง ดังที่ท่านนบีมุฮัมมัดได้กล่าวไว้ว่า "จะต้องไม่เบียดเบียนตนเองและผู้อื่น" ดังนั้น ขอให้คุณพ่อคุณแม่สบายใจ และพาลูกน้อยมาฉีดวัคซีนได้

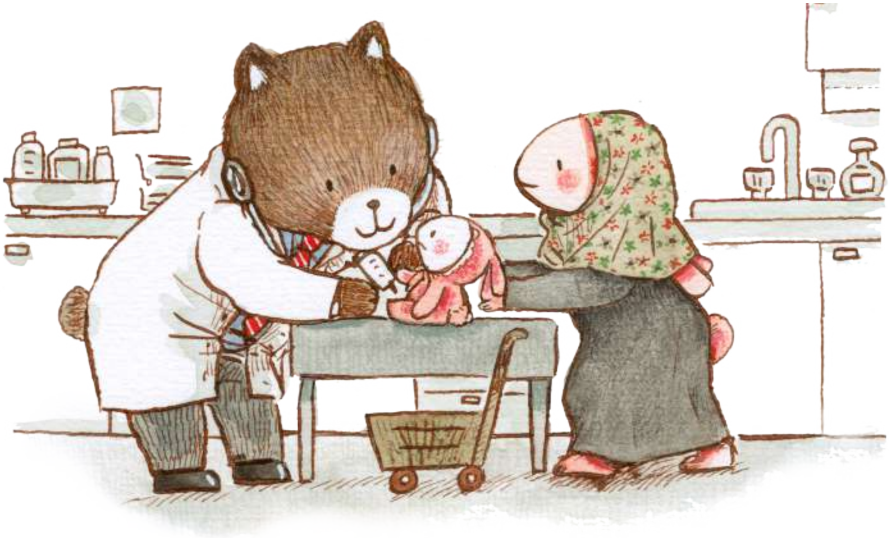

بوليهكه اورڠ مسلم منداڤت سونتيقن ۏكسين ؟

”كأدأن ضرورة داڤت مڠحلالكن ڤركارا يڠ حرام“

ڤڠرتين دري حكوم رخصة (كلوڠݢرن) دالم كأدأن ضرورة

مڠيكوت ڤڠرتين دري سورة البقرة : ايات 173

سكارڠ ترداڤت ڤڠيعترافن درڤد ڤارا علماء اسلام، علموان مسلم دان شيخ الاسلام يڠ مروڤاكن ڤميمڤين باݢي اݢام اسلام د تايلاند بهاوا اورڠ مسلم بوليه منريما سونتيقن ۏكسين. مريك برڤنداڤت يڠ سلارس بهاوا ڤوونتيقن ۏكسين اداله ڤنجاݢأن توبوه بادن يڠ دبريكن اوليه توهن مڠيكوت اجرنڽ. تيدق منريما سونتيقن ۏكسين سلأين درڤد مپاكيتي ديري سنديري، اي جوݢ مپاكيتي اورڠ لائين جك كيت منجادي اورڠ يڠ مريبقكن ڤپاكيت. اين دأڠݢڤ ساتو ڤرلاكوان دوسا مڠيكوت سبدا نبي محمد صلى الله عليه وسلم ” جاڠن مپاكيتي ديري سنديري دان اورڠ لأين.“ اوليه سبب ايت، دهارڤكن ايبو باڤ براس سنڠ هاتي دان ممباوا انق٢ اونتوق منداڤت سونتيقن ۏكسين.

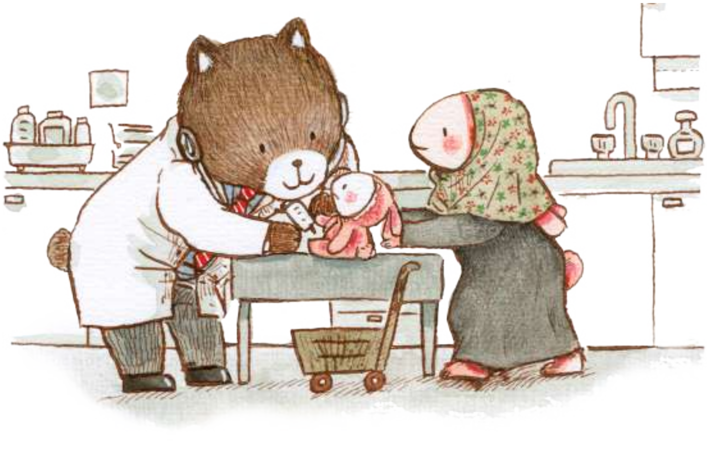

คุณหมอชวนคุย

ผศ.นพ.เทอดพงศ์ ทองศรีราช

วัคซีนคืออะไร

วัคซีนคือ ผลิตภัณฑ์ชีวภาพที่ประกอบด้วยเชื้อไวรัส หรือแบคทีเรียไม่มีชีวิต หรือทำให้มีฤทธิ์อ่อนจนไม่สามารถก่อโรคได้ ใช้กระตุ้นให้ร่างกายสร้างภูมิคุ้มกันโรค ซึ่งเกิดจากเชื้อนั้น ๆ โดยให้โดยการฉีดหรือหยอดทางปาก

ทำไมทุกคนควรได้รับวัคซีน

เมื่อแรกเกิด เด็กทุกคนจะมีภูมิต้านทานต่ำ เสี่ยงต่อโรคระบาดต่าง ๆ ที่อาจเกิดขึ้นได้ เช่น โปลิโอ คอตีบ ไอกรน บาดทะยัก หัด ซึ่งเชื้อเหล่านี้สามารถแพร่กระจายได้ง่าย ผู้ป่วยที่ติดเชื้อ คอตีบ ไอกรน หรือหัด อาจมีอาการรุนแรงมากถึงขั้นเสียชีวิตได้ แต่สามารถมีภูมิต้านทานโรคเหล่านี้ โดยการรับวัคซีนซึ่งมีประสิทธิภาพในการป้องกันโรคได้ดี และช่วยลดความรุนแรงของโรคเหล่านี้ได้

จะรับวัคซีนได้ที่ไหน

ผู้ปกครองสามารถพาบุตรหลานไปฉีดวัคซีนพื้นฐาน หรือวัคซีนจำเป็น ได้แก่ วัณโรค ตับอักเสบบี คอตีบ-บาดทะยัก-ไอกรน วัคซีนฮิบ วัคซีนโปลิโอ วัคซีนหัด-หัดเยอรมัน-คางทูม วัคซีนไข้สมองอักเสบเจอี ได้ฟรี ที่โรงพยาบาลของรัฐหรือโรงพยาบาลส่งเสริมสุขภาพประจำตำบล (รพ.สต.) ทั่วประเทศ ส่วนวัคซีนอื่น ๆ เช่น วัคซีนไข้หวัดใหญ่ วัคซีนสุกใส วัคซีนไอพีดี วัคซีนตับอักเสบเอ สามารถฉีดเสริมได้ เพื่อเพิ่มภูมิคุ้มกันร่างกายโดยมีค่าใช้จ่ายเพิ่มเติม

วัคซีนมีส่วนผสมจากสุกรจริงหรือไม่

ปัจจุบันวัคซีนพื้นฐานสำหรับเด็กในแผนการสร้างเสริมภูมิคุ้มกันโรคของกระทรวงสาธารณสุขของไทย **ไม่มีวัคซีนที่ใช้เอนไซม์ทริปซิน หรือ ใช้เจลาตินจากสุกร แล้ว**

การสร้างภูมิคุ้มกันโรคในมุมมองของอิสลาม

การป้องกันโรคเป็นหนึ่งในมาตรฐานของการดูแลสุขภาพ มี อายะฮอัลกุรอาน และ หะดีษ หลายบท ที่ให้ความชัดเจนในเรื่อง "**การป้องกันดีกว่าการรักษา**" การสร้างภูมิคุ้มกันโรคจึงมีความสอดคล้องกับหลักการอิสลามที่ว่า "**ปิดกั้นทุกหนทางที่จะทำให้เกิดความเสียหาย**" ดังเช่นในซูเราะห์อัลมาอิดะห์ อายะห์ที่ ๓๒ ความว่า "**และหากใครคนใดได้ช่วยเหลือชีวิตหนึ่งชีวิตใด ก็ประหนึ่งว่าเขาได้ช่วยเหลือชีวิตทั้งมวล**"

ข้อควรระวังก่อนฉีดวัคซีน

๑. หากมีไข้ควรเลื่อนวันฉีดวัคซีนออกไปก่อน ถ้ามีอาการป่วยเล็กน้อย เช่น เป็นหวัดโดยไม่มีไข้ สามารถให้วัคซีนได้

๒. หากเพิ่งได้รับเลือด ผลิตภัณฑ์ของเลือด หรือ อิมมูโนโกลบุลิน ควรเลื่อนการฉีดวัคซีนชนิดจำเป็น (เช่น วัคซีนหัด-หัดเยอรมัน-คางทูม วัคซีนไข้สมองอักเสบเจอี) ออกไปก่อน เนื่องจากวัคซีนอาจไม่ได้ผลหรือกระตุ้นภูมิคุ้มกันได้ไม่ดี

๓. หากแพ้วัคซีนหรือส่วนประกอบของวัคซีน ควรหลีกเลี่ยงการให้วัคซีนนั้น

๔. ผู้ที่แพ้ไข่ สามารถรับวัคซีนโรคหัดได้

๕. ไม่ควรให้วัคซีนเชื้อเป็นในหญิงมีครรภ์ และ ผู้ที่มีภูมิคุ้มกันบกพร่อง เช่น ได้ยากดภูมิต้านทาน

๖. กรณีไม่ได้มารับวัคซีนตามกำหนด ไม่จำเป็นต้องเริ่มต้นฉีดใหม่ ไม่ว่าจะเว้นช่วงห่างไปนานเท่าใด ให้นับต่อจากวัคซีนครั้งก่อนได้ (ยกเว้นวัคซีนพิษสุนัขบ้า)

๗. ผู้ที่สัมผัสโรคหัด สามารถเข้ารับวัคซีนป้องกันหลังสัมผัสโรคได้ หากสัมผัสโรคมาไม่เกิน ๓ วัน

๘. หลังได้รับวัคซีนป้องกันโรคคอตีบ ไอกรน บาดทะยัก เด็กบางคนอาจมีไข้ได้ แต่จะหายใน ๑-๒ วัน ควรเช็ดตัวและให้ยาลดไข้

دوكتور اجق بربوال

اسيستن ڤروفيسسور دوكتور تودڤوڠ طاڠسيرات

اڤاكه ايت ۏكسين؟

ۏكسين اياله ڤرودوق بيولوݢي يڠ ترديري درڤد كومن ۏيروس اتاو بكتيريا يڠ تله ماتي اتاو دڤرلمهكن سهيڠݢ تيدق داڤت مڠعاقبتكن ڤپاكيت. ۏكسين دݢوناكن اونتوق مرڠسڠ بادن سوڤاي ممبينا داي تاهن ڤپاكيت يڠ دعاقبتكن كومن٢ ترتنتو دڠن چارا دسونتيق اتاو دتيتيسكن كدالم مولوت.

مڠاڤاكه ستياڤ اورڠ ڤرلو منداڤت ۏكسين؟

سماس دلاهيركن، ستياڤ اورڠ كانق٢ ممڤوڽاٴي داي تاهن يڠ رنده، بركموڠكينن دجڠكيتي ڤلباݢاي ڤپاكيت برجڠكيت سڤرتي ڤوليو، ايسق، باتوق كوڠكول، تيتانوس (بدتايق) دان چمڤق. كومن٢ ترسبوت داڤت مريبق دڠن سنڠ. ڤساكيت يڠ منداڤت كومن ايسق، باتوق كوڠكول اتاو چمڤق موڠكين ممڤوڽاٴي كچاچتن يڠ ترووق سهيڠݢ ماتي. نامون كانق٢ بوليه منداڤت داي تاهن باݢي ڤپاكيت ترسبوت دڠن چارا منريما ۏكسين يڠ بركسن اونتوق مڠيلقكن ڤپاكيت دان مڠورڠكن كموضرتن ڤپاكيت ترسبوت.

د ماناكه تمڤت منريما ۏكسين؟

ايبو باڤ بوليه ممباوا انق - چوچواونتوق منداڤت ۏكسيس اساس اتاو ۏكسين يڠ دڤرلوكن سڤرتي باتوق كريڠ، رادڠ هاتي- ب، ايسق، تيتانوس (بدتايق) ، باتوق كوڠكول، ۏكسين هيب، ۏكسين ڤوليو، ۏكسين چمڤق- چمڤق جرمن- بڠكق ڤيڤي دان ۏكسين دمم رادڠ اوتق جي.اي.

دڠن ڤرچوما دهوسڤيتل كراجأن اتاو هوسڤيتل ممبينا كصيحتن د مقيم (اناماي) د سلوروه نڬارا. باڬي ۏكسين لأين سڤرتي ۏكسين سلسما، ۏكسين چاچر اير، ۏكسين أي. ڤي. دي.، ۏكسين رادڠ هاتي إي. بوليه جوك دداڤتكن اونتوق منمبه داي تاهن توبوه بادن دڠن دكناكن بايرن تمبهن.

بتولكه ۏكسين ممڤوڤاءي باهن چمڤورن دري بابي ؟

سكارڠ ۏكسين اساس اونتوق كانق٢ دالم رنچڠن ڤمبيناءن داي تاهن اونتوق مڠيلقكن ڤڽاكيت باڬي كمنترين كصيحتن تايلاند **سوده تيدق اد لاڬي ۏكسين يڠ مڠڬوناكن اينزيم تريڤسين اتاو جيلاتين دري بابي.**

ڤمبيناءن داي تاهن دالم كاچ مات اسلام

ڤڠيلقن ڤڽاكيت مروڤاكن ساتو ڤياواي باڬي ڤنجاڬاءن كصيحتن. ترداڤت بابق ايات القرءان دان حديث يڠ منجلسكن تنتڠ " ڤڠيلقن لبيه بأيق درڤد راوتن". مك ڤمبيناءن داي تاهن ڤڽاكيت سلارس دڠن اجرن اسلام بهاوا " توتوڤ ستياڤ جالن يڠ موڠكين مڠعاقبتكن كروسقن" سڤرتي يڠ دسبوت دالم سورة المائدة، ايات 32 يڠ برارتي " دان سسياڤا يڠ ميلامتكن ڽاوا سسأورڠ، دي دأڠڬڤ ميلامتكن سمووا اورڠ. "

ڤريغتن سبلوم منداڤت سونتيقن ۏكسين

1. كالاو دمم، تغكوهكن دهولوهاري منريما ۏكسين. تتاڤي كالاو ترداڤت ݢجالا يڠ ريڠن سڤرتي سلسما تنڤا دمم، مك بوليه منريما ۏكسين.

2. جك بارو منريما داره، ڤرودوق درڤد داره اتاوهيموݢلوبولين، سڤاتوتڽ تڠݢوه دهولو سونتيقن ۏكسين يڠ دڤرلوكن (سڤرتي ۏكسين چمڤق- چمڤق جرمن- بڠكق ڤيڤي، ۏكسين دمم رادڠ اوتق جي.اي.) كران ۏكسين موڠكين جادي تيدق بركسن اتاو تيدق داڤت مرڠسڠ داي تاهن دڠن باٴيق.

3. جك اله ۏكسين اتاو باهن چمڤورنڽ، ايلقكن ڤنريماٴن ۏكسين ايت.

4. اورڠ يڠ اله تلور بوليه منريما ۏكسين چمڤق.

5. تيدق ڤاتوت ممبريكن ۏكسين كومن هيدوف كڤد ڤرمڤوان يڠ سدڠ مڠندوڠ دان اورڠ يڠ ممڤوڽاٴي كروسقن داي تاهن سڤرتي ماكن اوبت منكن داي تاهن.

6. سكيراڽ تيدق داڤت داتڠ منريما ۏكسين ڤد تڠݢل يڠ دتنتوكن، تيدق ڤرلو مولاكن ڤروسيس ڤنريماٴن ۏكسين سمولا، تيدق دكيرا براڤ لاما سكالي ڤون تيمڤوهڽ، جومله سونتيقن ۏكسين هندقله دهيتوڠ سچارا برتروسن دري سونتيقن ۏكسين سبلومڽ (كچوالي ۏكسين انجيڠ ݢيلا).

7. اورڠ يڠ مينتوه كومن ڤاكيت چمڤق بوليه منداڤت ۏكسين سلڤس مينتوه كومن ايت جك دي مينتوه كومن ايت تيدق لبيه درڤد 3 هاري

8. سلڤس منداڤت ۏكسين ڤاكيت ايسق، باتوق كوڠكول، تيتانوس (بدتايق) ، سستڠه كانق٢ موڠكين منجادي دمم، تتاڤي اكن سمبوه دالم ماس 1-2 هاري، ڤاتوت مڠلق بادن دان ممبري اوبت دمم.

ทำไมเด็กที่ป่วยเป็นโรคหัดจึงอาจมีอาการรุนแรงมากจนเสียชีวิต

ไวรัสหัด เป็นไวรัสที่ติดต่อกันได้ง่ายมากจากการหายใจเอาอากาศที่มีเชื้อหัดเข้าไป ซึ่งเชื้อหัดจะอยู่ในสารคัดหลั่งของผู้ป่วย เช่น น้ำมูก น้ำลาย ทำให้แพร่กระจายสู่ผู้อื่นได้ง่าย หากเด็กที่ไม่มีภูมิต่อโรคนี้ได้สัมผัสโรคหัด จะมีโอกาสติดเชื้อได้ถึง ๙๐%

ไวรัสหัดจะเข้าไปทำลายเยื่อบุของทางเดินหายใจ ทำให้ภูมิคุ้มกันของร่างกายอ่อนแอลง และติดเชื้อแบคทีเรียแทรกซ้อนตามมาได้ เช่น ปอดอักเสบจากติดเชื้อแบคทีเรีย จากข้อมูลพบว่า ปอดอักเสบรุนแรงเป็นสาเหตุที่สำคัญในการเสียชีวิตของผู้ป่วยโรคหัด

ภาวะแทรกซ้อนของโรคหัดมีอะไรบ้าง

อุจจาระร่วง ปอดบวม/ปอดอักเสบ กล่องเสียงและหลอดลมอักเสบ หูชั้นกลางอักเสบ สมองอักเสบ ตับอักเสบ เกล็ดเลือดต่ำ และภาวะติดเชื้อแบคทีเรียแทรกซ้อน เช่น ปอดอักเสบรุนแรงจากเชื้อนิวโมคอคคัส

โรคหัดมีการรักษาอย่างไร

ปัจจุบันยังไม่มียาต้านไวรัสหัด จึงยังไม่มีการรักษาจำเพาะสำหรับโรคหัด ดังนั้น การรักษาหลักคือการรักษาตามอาการ เช่น ให้สารน้ำ ยาลดไข้พาราเซตามอล ยาแก้ไอ และให้ยาปฏิชีวนะกรณีมีติดเชื้อแบคทีเรียแทรกซ้อน โดยผู้ป่วยหัด**ทุกรายต้องได้รับวิตามิน A** เนื่องจากมีข้อมูลที่แสดงว่า การให้วิตามิน A ช่วยลดอัตราการเสียชีวิตและภาวะแทรกซ้อนในผู้ป่วยโรคหัดได้ เนื่องจากวิตามิน A จะช่วยเสริมความแข็งแรงของเยื่อบุทางเดินหายใจ และเสริมภูมิต้านทานของร่างกายในการต่อสู้กับไวรัสหัดได้

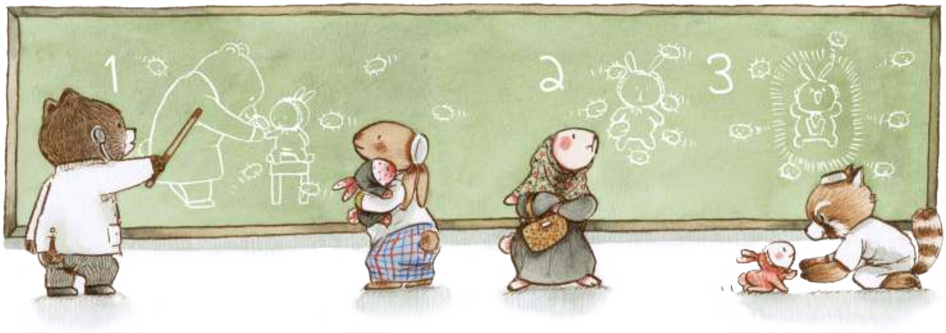

مغاڤاكه كانق٢ يغ مغهيدڤي چمڤق موغكين ممڤوڤاءي كَجالا يغ تروق سهيغك ممباوا كڤد ماتي؟

ۏيروس چمڤق اياله ۏيروس يغ برجغكيت دغن موده ملالوءي سدوتن اودارا يغ ممڤوڤاءي كومن چمڤق. كومن چمڤق برادا دالم ريمبيسن چچاءير ڤساكيت سڤرتي هيڠوس، اءير ليور يغ ميببكن كومن داڤت مريبق دغن سنڠ. جك كانق٢ تيدق ممڤوڤاءي داي تاهن باكَي ڤپاكيت اين ترسنتوه دغن كومن ڤپاكيت چمڤق، دي بركموغكينن 90% دجڠكيتي ڤپاكيت ايت.

ۏيروس چمڤق اكن ماسوق مروسقكن تيسو سالورن ڤرنافسن، ميببكن داي تاهن سماكين لمه دان دجڠكيتي بكتيريا سڤرتي رادڠ ڤارو٢ دري جڠكيتن بكتيريا. دداڤتي بهاوا رادڠ ڤارو٢ يڠ تروق اداله ڤونچا اوتام باكَي كماتين ڤساكيت چمڤق.

اڤاكه كومڤليكاسي (ڤپاكيت سمڤيڠن) باكَي ڤپاكيت چمڤق؟

چيريت- بيريت، بڠكق ڤارو٢ / رادڠ ڤارو٢، رادڠ كوتق سوارا دان سالورن اودارا، رادڠ تليڠالاڤيسن ڤرتڠهن، رادڠ اوتق، رادڠ هاتي، ككورڠن سيسيق داره دان كومڤليكاسي جڠكيتن بكتيريا سڤرتي رادڠ ڤارو٢ يڠ تروق عاقبة كومن نيوموكوككس .

باڬايماناكه چارا مڠوبتي ڤپاكيت چمڤق؟

سكارڠ ماسيه بلوم اد اوبت انتي- ۏيروس (اوبت لاون ۏيروس) چمڤق، مك تياد راوتن ڤپاكيت چمڤق سچارا خاص. اوليه سبب ايت راوتن اوتام اياله راوتن مڠيكوت كَجالا ڤپاكيت سڤرتي ممبريكن باهن اير، اوبت تاهن ساكيت ڤاراسيتامول، اوبت باتوق دان اوبت انتي-بيوتيك

جك ترداڤت كومڤليكاسي جڠكيتن بكتيريا. **ستياڤ ڤساكيت چمڨق مستي منداڤت ۏيتامين** A كران دداڤتي بهاوا ۏيتامين A داڤت ممبنتو مڠورڠكن قدر كماتين دان كومڤليكاسي باڬي ڤساكيت چمڨق اوليه سبب ۏيتامين اي داڤت ممبنتو مڠواتكن تيسو سالورن ڤرنافسن دان داي تاهن اونتوق مالاون ۏيروس چمڨق.

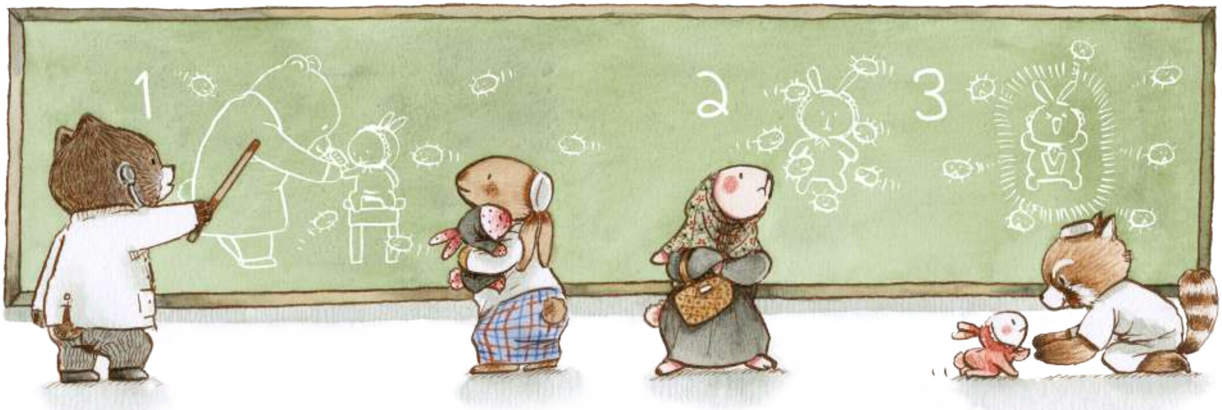

ตารางการให้วัคซีนในเด็กไทย
แนะนำโดยสมาคมโรคติดเชื้อในเด็กแห่งประเทศไทย พ.ศ.๒๕๖๒

วัคซีนจำเป็นที่ต้องให้กับเด็กทุกคน											
วัคซีน	แรกเกิด	๑ เดือน	๒ เดือน	๔ เดือน	๖ เดือน	๙-๑๒ เดือน	๑๘ เดือน	๒ ปี	๒.๕ ปี	๔-๖ ปี	๑๑-๑๒ ปี
บีซีจี (BCG)	BCG										
ตับอักเสบบี (HBV)	HBV1	HBV2	DTwP-HB-Hib1	DTwP-HB-Hib2	DTwP-HB-Hib3						
คอตีบ-บาดทะยัก-ไอกรนชนิดทั้งเซลล์ (DTwP)							DTwP กระตุ้น ๑			DTwP กระตุ้น ๒	Td และทุก ๑๐ ปี
ฮิบ (Hib)											
โปลิโอชนิดกิน (OPV)			OPV1	OPV2+ IPV	OPV3		OPV4			OPV5	
หัด-หัดเยอรมัน-คางทูม (MMR)						MMR1		MMR2			
ไข้สมองอักเสบเจอี (Live JE)						JE1		JE2			
ไข้หวัดใหญ่ (influenza)					Influenza ให้ ๒ เข็ม ห่างกัน ๑ เดือน ในครั้งแรก						
เอชพีวี (HPV)											ด.ญ ป๕ ฉีด ๒ เข็ม ห่างกัน ๖-๑๒ เดือน

เพลง วัคซีน สดใส

ทำนอง เพลงละหมาด ๕ เวลา

เช้าตรู่ ฉันสดใส จะไปโรงเรียน
ไปโรงเรียน พบเพื่อนมากมาย
เพื่อน ๆ รอบกาย มีตุ่มมากมาย
เพื่อน ๆ รอบกาย มีตุ่มมากมาย
ตาแดง ตัวลาย หาหมอเร็วไว

หมอบอกว่าต้องฉีดวัคซีน
ฉีดวัคซีน ป้องกันโรคหัด
ไม่ต้องกลัวนะ ไม่เจ็บหรอกจ้ะ
ไม่ต้องกลัวนะ ไม่เจ็บหรอกจ้ะ
นบีกล่าวไว้ ต้องรักษากาย

จงจำไว้ ต้องฉีดวัคซีน
ฉีดวัคซีน ป้องกันโรคหัด
มาฉีดกันนะ ฮาลาลด้วยนะ
มาฉีดกันนะ ฮาลาลด้วยนะ
ทุกคนฉีดแล้ว สดใส แข็งแรง

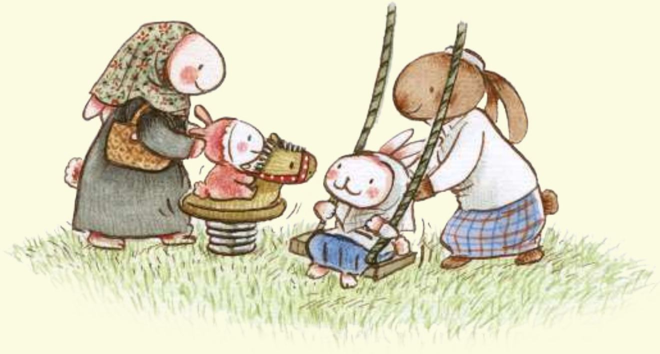

อานีส
انيس
เป็นหัด
مڠهيدڤي چمڤق

คณะผู้จัดทำ ผศ.นพ.เทอดพงศ์ ทองศรีราช และชนาธิป ไทห์ ณัฐกมล พงษ์พูล เตชัส รัตนานุสนธิ์ ธนกร ปรีชาสุชาติ ธนธรณ์ สำเร็จ นิลุบล อุดมกิระทักษ์ ปานรดา สมิทธิฐิติ สวรรณยา ตะบูนพงษ์ [นักศึกษาชั้นปีที่ ๔ (พ.ศ. ๒๕๖๒)] คณะแพทยศาสตร์ มหาวิทยาลัยสงขลานครินทร์

คณาจารย์ที่ปรึกษา

อ.พญ.พุทธิชาติ ขันตี นพ.รพีพัฒน์ เทวมิตร์ อาจารย์ประจำภาควิชากุมารเวชศาสตร์ มหาวิทยาลัยสงขลานครินทร์

ดร.อัสมัน แตอาลี รองคณบดีฝ่ายบริการวิชาการและวิเทศสัมพันธ์ วิทยาลัยอิสลามศึกษา มหาวิทยาลัยสงขลานครินทร์ วิทยาเขตปัตตานี

นายรอมฎอน หะสาเมาะ รองประธานมูลนิธิเบิกฟ้าอิสลาม สำนักจุฬาราชมนตรี ศูนย์ภาคใต้

นางสาวมารีนา หมัดอะด้ำ นักวิชาการศึกษา สถาบันฮาลาล มหาวิทยาลัยสงขลานครินทร์

ภาพ สวนีย์ พรวิศวารักษกูล **บรรณาธิการ** ระพีพรรณ พัฒนาเวช สุดใจ พรหมเกิด มัจญ์ดัน มาโซ อัสมา นาคเสวี

กองบรรณาธิการ หทัยรัตน์ พันตาวงษ์ นันทพร ณ พัทลุง สิราภรณ์ ชาวหน้าไม้ นิตยา หอมหวาน ปนัดดา สังฆทิพย์ ตัรมีซี อาหามะ จันทิมา อินจร ธัญนรี ทองชุม นิศารัตน์ อำนาจอนันต์ สุธาทิพย์ สรวยล้ำ **ผู้แปล** : แวมายิ ปารามัล

ออกแบบและจัดหน้า น้ำฝน อับดุลเลาะ หลงสะ **ประสานงานการผลิต** สิริวัลย์ เรืองสุรัตน์ มัจญ์ดัน มาโซ

พิมพ์ครั้งที่ ๑ : ตุลาคม ๒๕๖๒ จำนวน ๖,๐๐๐ เล่ม พิมพ์ครั้งที่ ๒ (ภาษาไทย-มลายู) : พฤษภาคม ๒๕๖๓ จำนวนพิมพ์ : ๑,๐๐๐ เล่ม

จัดพิมพ์และเผยแพร่โดย องค์การช่วยเหลือเด็ก (Save the Children)

พิมพ์ที่ : บริษัท แปลน พริ้นท์ติ้ง จำกัด **โทรศัพท์** : ๐ ๒๒๗๗ ๒๒๒๒

ISBN : 978-616-93372-8-7

แผนงานสร้างเสริมวัฒนธรรมการอ่าน บริหารงานโดย "มูลนิธิสร้างเสริมวัฒนธรรมการอ่าน" ได้รับการสนับสนุนจากสำนักงานกองทุนสนับสนุนการสร้างเสริมสุขภาพ (สสส.) ดำเนินงานประสานกลไก นโยบาย และปัจจัยขยายผลจากทั้งภาครัฐ ภาคประชาสังคม และภาคเอกชน ให้เอื้อต่อการขับเคลื่อนการสร้างเสริมพฤติกรรมและวัฒนธรรมการอ่านให้เข้าถึงเด็ก เยาวชน และครอบครัว โดยเฉพาะกลุ่มที่ขาดโอกาสในการเข้าถึงหนังสือ และกลุ่มที่มีความต้องการพิเศษ

ร่วมสนับสนุนการขับเคลื่อนนโยบาย โครงการ และกิจกรรมสร้างเสริมวัฒนธรรมการอ่านเพื่อสร้างสังคมสุขภาวะได้ที่

มูลนิธิสร้างเสริมวัฒนธรรมการอ่าน

๔๒๔ หมู่บ้านเงาไม้ ซอยจรัญสนิทวงศ์ ๖๗ แยก ๓ ถนนจรัญสนิทวงศ์ แขวงบางพลัด เขตบางพลัด กรุงเทพ ฯ ๑๐๗๐๐

โทรศัพท์ : ๐ ๒๔๒๔ ๕๖๑๖ **โทรสาร** : ๐ ๒๘๘๑ ๑๘๗๗ **Email** : happy2reading@gmail.com **Website** : www.happyreading.in.th

http://www.facebook.com/Happyreadingnews (วัฒนธรรมการอ่าน Happyreading)
